# The strawberry genome: a complicated past and promising future

**DOI:** 10.1038/s41438-019-0181-z

**Published:** 2019-08-21

**Authors:** Kevin M. Folta, Christopher R. Barbey

**Affiliations:** Horticultural Sciences Department, University of Florida, Gainesville, FL 32611 USA

**Keywords:** Polyploidy in plants, Plant evolution

Within the juicy red flesh of the commercial strawberry lies a deep history that spans at least three continents and hundreds of thousands of years. The genetic heritage of this prized dessert fruit was crafted by nature, with its modern improvement driven by the indigenous Mapuche people of South America, a seafaring a French spy, and a plant-loving teenager that gathered fruits to present to a king. The many stories that punctuate the history of the modern strawberry relate to its contemporary cultivation as well as origins that span a significant portion of the globe^1,2^. The evidence of this rich history is locked away within the chromosomes of every cell, each archiving a complicated story that speaks both of strawberry’s historical origins and genetic potential for future production.

The complete genetic storybook of the commercial strawberry has remained a secret to researchers, until now. Compared to other economically important plant species, commercial strawberry was slow to garner molecular attention, with only 58 small and mostly mis-annotated DNA sequences present in public databases in 2003. Bits and pieces were gathered through the next few years^3,4^, with a genome of a simple diploid *Fragaria vesca* strawberry eventually published in 2011^5^. At the time it represented the 12th plant genome sequenced, and the first to be sequenced and assembled using solely short-read technology. The commercial strawberry is octoploid, and some genome sequence has been reported by several groups^6,7^ that used a variety of short read technologies and genetic strategies to derive substantial, yet incomplete coverage and assembly.

These avenues of progress set the table for the eventual publication of the octoploid strawberry sequence in February of 2019^8^. The team obtained the 813.4 Mb cultivated commercial strawberry sequence from the California cultivar “Camarosa” and it reveals the reticulate roots of a curious species. The cultivated strawberry is known scientifically as *Fragaria* × *ananassa*, with the “×” reminding us that it is a hybrid borne of a human-facilitated, blind-date sexual connection between two distantly related New World species, *Fragaria virginiana* (from North America) and *Fragaria chiloensis* (from South America). The genomes of these two octoploid species contain the genetic artifacts of interspecies hybridization ferried forward likely because of the fitness of the resulting natural hybrid. The genetic evidence of connections across time and space reside in every single cell. This information has now been used to unravel strawberry’s complicated history, understand its current biology, and design molecular tools to speed future improvement.

## Unwinding the past

While the modern strawberry’s chromosome collection is genomically complex, its fundamental genome is one of the simplest among crop plants. The strawberry of commerce is octoploid (2*n* = 8× = 56; seven chromosome sets and eight chromosomes per set, 56 total), meaning that each cell contains remnants of four separate ancestral diploid subgenomes that underlie strawberry’s form and function. Examination of the origins of these four subgenomic complements began early in the 20th century, with study of meiotic pairing^9,10^, suggesting that an ancestor of the extant diploid species *F. vesca* was a contributor to the octoploid genome. Small hints of the identity of other subgenome donors came from genetic analyses and various reconstructions^11,12^, along with some molecular^13^ and cytological^14^ data that provided critical clues. Several of these avenues suggested that an ancestor of *F. iinumae* was at least one of the other subgenome donors.

As pointed out by Edger et al.^15^, the genome is an allopolyploid, arising from multiple rounds of gametic nonreductions and cross pollination events. Today the resulting subgenomes continue to behave as separate blueprints interpreted simultaneously to define the assembly and function of a common complex structure. The multiple-blueprint problem has hampered simple genetic analyses, as if a locus was mapped in a diploid genome, it may reside in any, or all, of the subgenomes that comprise the modern octoploid strawberry. To make matters worse, strawberry is highly heterozygous, making it difficult to distinguish between homoeologous and paralogous gene copies.

The recently published “Camarosa” sequence strengthened the evidence identifying the other genomic constituents in commercial strawberry. The work agrees with earlier findings that *F. iinumae* and *F. vesca* are closest descendants to two of the four subgenome donors. But there is great diversity within *F. vesca*, a species that covers the northern hemisphere. The authors were able to narrow down the subspecies to *F. bracheata*, consistent with other findings that suggest this genotype contributed the maternal genome^16^. The authors identified genome sequence most closely resembling modern day *F. nipponica*, implying that ancestors of this species (that were sympatric with *F. iinumae* in Japan) gave rise to proximal tetraploid genotypes in neighboring China. The analysis also identified sequence most resembling the Eurasian genotype *F. viridis*, supporting some previous hypotheses^17^.

## Unveiling genomic structure and function

The complete sequence of the octoploid strawberry provides insight into the elements that shape functional subgenome interactions. The commercial strawberry provides an ideal model to study hybridization-induced genomic shock. Massive genome-remodeling takes place when the genomes of different species are forced to cohabitate inside a single nucleus. The work by Edger et al.^8^ shows that the subgenome derived from the diploid *F. vesca* dominates in gene number and gene expression. The *F. vesca* genome was the most recent to be added to the octoploid strawberry, however, its dominant expression is likely not due to its recent incorporation.

Gene expression is known to be suppressed by proximity to transposable elements (TEs)^18,19^, and the genome of *F. vesca* maintains relatively low-TE content^5^. This finding provides a likely explanation of why *F. vesca* came to quickly dominate gene expression among resident subgenomes. In the 1 million years since the chance formation of the hybrid octoploid genome, vestigial portions of the non-*F. vesca* subgenomes were eventually supplanted by *F. vesca* sequences^8^. Several mechanisms for this phenomenon have been identified^18^. Aberrant crossover between strawberry’s highly similar subgenomes during meiosis led to unequal chromosomal exchanges and gene conversion, as has been reported in coffee^20^. In a-thousand-millennias-worth of meioses, strawberry’s subgenomes became more and more similar. This similarity is part of what made distinguishing the four subgenomes of strawberry so difficult using traditional methods. It is the basis for what has made connecting traits to specific genes so challenging.

Such biased exchanges during chromosome recombination has been reported in other allopolyploids, and is thought to be connected to subgenome dominance. Evidence supporting his hypothesis comes from the higher prevalence of *F. vesca* R-genes within the nondominant subgenomes. Again, the elucidation of the complete octoploid sequence allows scientists to begin to piece together how the dominant subgenome shaped the genome as a whole under the process of selection.

## Guiding the future

The octoploid strawberry genome sequence will aid basic research and commercial strawberry improvement. Previous octoploid QTL results were often examined in reference to the *F. vesca* genome, which by definition tells only part of the story. While *F. vesca* is important to the overall character of commercial strawberry, and the preponderance of expressed genes are *F. vesca-*like, we know now that it is common for these *F. vesca*-like genes to actually reside within other subgenomes^8^. Therefore, the *F. vesca* genome likely may have been less useful as an octoploid strawberry reference^21,22^ than previously assumed. The octoploid genome sequence will solve these deficits via the capacity to integrate QTL analysis with octoploid genomics. Mapping subgenomic markers can be greatly improved using the octoploid genome, as only about a third of iStraw35 markers (the most popular genotyping platform)^23^ are currently incorporated into a genetic map. The octoploid genome can therefore be used to improve the resolution of existing QTL and provide a clearer roadmap for the identification of causal variants.

This is not the only way the octoploid genome can breathe new life into old data. Raw RNAseq octoploid short reads, previously limited to low-resolution *de novo* assembly analysis, can be reassembled based on the octoploid reference to enormously enhance the level of resolution. Such reanalysis is underway at strawberry research institutions around the globe. This re-analysis will allow for the novel discrimination of alleles and homoeologs in commercial octoploid strawberry, and opens the door to integrative multi-omics analysis with sensitivity to the octoploid level.

The complete octoploid strawberry sequence provides an accounting of the genes that may govern important agricultural traits. A great example is the suite of R-genes, or genes relevant to plant disease resistance. Edger and colleagues identify the various subtypes and note their considerable expansion over the diploid *F. vesca* genome alone. These findings are of great interest, as few resistance genes have been characterized in strawberry, and they may represent new tools for molecular marker development that will allow breeders to coalesce multiple resistance genes into a single genetic background. Such advances would go a long way to increasing sustainable production of strawberry.

The genome also provides an anchor to tie in years of functional data obtained by transgenic or transient-expression analyses. The molecular basis for traits in strawberry have mostly been identified using these techniques, yet few of these published reports describe natural sources of genetic variation that can be exploited by breeders. Selection of eQTL-based markers may help translate an existing body of molecular work into widely applicable markers that can hasten variety improvement. In addition, an octoploid genome sequence offers an obvious advance towards defining gene editing targets that are specific to a desired single locus. The octoploid genome sequence is also a starting point to dissect functional aspects of variety-specific attributes, and enables new approaches such as capture-based sequencing.

Finally, the octoploid genome represents a crucial first step in developing the strawberry pan-genome—it will serve as the reference for the compendium of octoploid genomes that matter throughout the world. Cultivars and advanced selections from many breeding programs now can be examined with the meaningful molecular scrutiny of a subgenome location. The fine relationships between the wild octoploids and modern germplasm can be resolved, and potentially the wild accessions can provide a wealth of genetic resources to install new alleles for flavor, stress resilience and disease tolerance lost after centuries of intense annual selection.

## Conclusions

The publication of complete octoploid strawberry sequence has accomplished what most high quality genomic resources do—they enable more questions to be asked in a meaningful way. In this case the information resolves a centuries-old question of subgenome composition, provides an understanding of how those subgenomes are organized and interact, and reveals a complete parts list for how to make a much appreciated and nutritious fruit. The sequence data can now be put into work in practical breeding application to improve strawberry and increase the economic and environmental viability of its production.
